# CD73 and PD-L1 expression in WHO grade 1 and grade 2 meningiomas: an exploratory analysis of purinergic and immune-related profiles

**DOI:** 10.1007/s11060-026-05786-y

**Published:** 2026-09-14

**Authors:** João Victor Garcia de Souza, Kailane Paula Pretto, Laryssa Vitória Gomes da Silva, Brendha Aparecida Macedo Oenning, Chen Pin, Jerso Menegassi, Francini Franscescon, Matheus Bianchini Chimelo, Marcelo Lemos Vieira da Cunha, Débora Tavares de Resende e Silva

**Affiliations:** 1 Graduate Program in Biomedical Sciences, Federal University of Fronteira Sul (UFFS), Rodovia SC 484 - Km 02, Chapecó, CEP 89815-899 Santa Catarina Brazil; 2 Department of Nursing, Federal University of Fronteira Sul (UFFS), Chapecó, Santa Catarina Brazil; 3 Postgraduate Program in Biomedical Sciences, Universidade Federal da Fronteira Sul, Chapecó, Brazil; 4 School of Medicine, Universidade Católica de Pelotas, Pelotas, RS Brazil; 5 Residency Program in Pathology, Universidade Federal de Santa Maria, Santa Maria, RS Brazil; 6 Santa Catarina Society of Neurosurgery, Chapecó, Santa Catarina Brazil

**Keywords:** Meningioma, Purinergic system, CD73, PD-L1, Tumor microenvironment

## Abstract

**Background:**

Meningiomas are the most common primary central nervous system tumors and exhibit heterogeneous biological behavior across WHO grades. Emerging evidence suggests that purinergic signaling and immune-related pathways may contribute to meningioma biology.

**Objective:**

This study evaluated the expression and activity of CD39/ENTPD1 and CD73/NT5E, the expression of PD-L1/CD274, and the systemic inflammatory profile in patients undergoing surgical resection of meningiomas.

**Results:**

Forty patients were included, with specific subgroups available for molecular, immunohistochemical, enzymatic, and cytokine analyses. Molecular comparisons were restricted to WHO grade 1 and grade 2 tumors. NT5E/CD73 expression was higher in WHO grade 2 tumors at both mRNA and protein levels, whereas CD274/PD-L1 expression was lower. Peripheral CD39/CD73 enzymatic activity also differed between study groups, while plasma cytokines showed non-significant trends toward higher concentrations in patients with WHO grade 2 tumors.

**Conclusion:**

Given the cross-sectional design, small sample sizes for specific analyses, and the absence of direct measurements of adenosine signaling or longitudinal clinical outcomes, these findings should be interpreted as exploratory patterns associated with tumor grade rather than evidence of tumor progression or a causal mechanism of immune evasion. Overall, the results identify CD73 as a candidate biomarker associated with WHO grade, warranting validation in larger, longitudinal, and mechanistic studies.

## Clinical trial number

Not applicable.

## Introduction

Meningiomas are primary tumors affecting the meninges of the Central Nervous System (CNS), representing the most frequent tumors in this area. They originate from arachnoid cells located on the inner surface of the dura mater, accounting for approximately 36% of CNS tumors, with an incidence ratio of 2 women to 1 man, predominating in individuals over 65 years of age [1, 2].

According to the World Health Organization (WHO), the classification of malignancy or benignity of a brain tumor follows grading scales that consider factors such as malignancy, aggressiveness, and growth rate. WHO Grade 1 consists of tumors considered benign, which are possibly curable with surgery and have slow growth, for example, pilocytic astrocytoma. WHO Grade 2 tumors have similar characteristics to WHO grade 1, but with a greater chance of the tumor manifesting at a higher grade. WHO Grade 1 and 2 tumors are considered low-grade. High-grade tumors, which are levels 3 and 4, are malignant tumors with rapid and aggressive growth, such as glioblastoma multiforme [3].

Inflammation is an essential component of the innate immune response, normally activated as a defense mechanism against infections, trauma, or other forms of tissue damage. However, when persistent or dysregulated, it can contribute to chronic pathological processes, including the development and progression of neoplasms. The association between inflammation and cancer was initially proposed by Virchow in the 19th century and has since been extensively studied [4].

In this context, the Purinergic System (PS) plays a fundamental role in cellular homeostasis and intra- and extracellular signaling, acting intrinsically in the CNS and is evidenced in an altered manner in various tumor types. It is composed of nucleotides such as Adenosine Triphosphate (ATP), which is hydrolyzed into Adenosine Diphosphate (ADP), Adenosine Monophosphate (AMP), and Adenosine Monophosphate (ADO), acting through binding to different types of receptors, generating specific cellular responses, excitatory or inhibitory [5, 6]. Its regulation is mediated by the action of cluster of differentiation (CD39) enzymes (ecto-nucleoside triphosphate 17 diphosphohydrolase 1, E-NTPDase1) and cluster of differentiation 73 (CD73) (ecto-5’-nucleotidase, Ecto5’NTase), which have been described as intrinsic components of the tumor microenvironment (TME) [7, 8]. In addition to the CD39-CD73 axis, adenosine deaminase (ADA) acts by removing interstitial ADO [9, 10].

The tumor microenvironment (TME) is a cellular and molecular ecosystem that surrounds and interacts with tumor cells, directly influencing their survival, growth, and immune evasion capacity. It is composed of a diversity of non-tumor cells, such as cancer-associated fibroblasts, endothelial cells, innate and adaptive immune cells, as well as components of the extracellular matrix and soluble molecules, such as cytokines, growth factors, enzymes, and immunosuppressive metabolites, such as adenosine [4, 11].

This network of dynamic interactions modulates angiogenesis, inflammation, local metabolism, and treatment response, making the TME an important determinant of tumor aggressiveness and therapeutic resistance [12]. In this context, understanding the immunological and metabolic mechanisms of the tumor microenvironment has driven the development of new therapeutic strategies, especially immunotherapies that target not only neoplastic cells but also the elements that support and protect the tumor [13].

Although SP components are widely studied in other CNS tumors, such as gliomas and glioblastomas, the literature on their role in meningiomas is still scarce. Because they are, in most cases, benign tumors, meningiomas tend to receive less attention regarding the role of SP in their development and progression. However, the fact that they are the most common primary tumor type reinforces the need for further study in this field. Understanding the molecular mechanisms involved in the pathophysiology of meningiomas, such as SP markers, is crucial for understanding the biological behavior of these tumors, to generate advances in improving patient care.

## Methodology

### Ethical aspects

The study followed current ethical guidelines and was approved by the Research Ethics Committee with Human Beings of the Federal University of the Southern Frontier (UFFS), under opinion number 5.975.092 and CAAE number 65163722.0.0000.5564. All participants signed the Informed Consent Form (ICF).

### Design, location, and sample

**Fig. 1:**
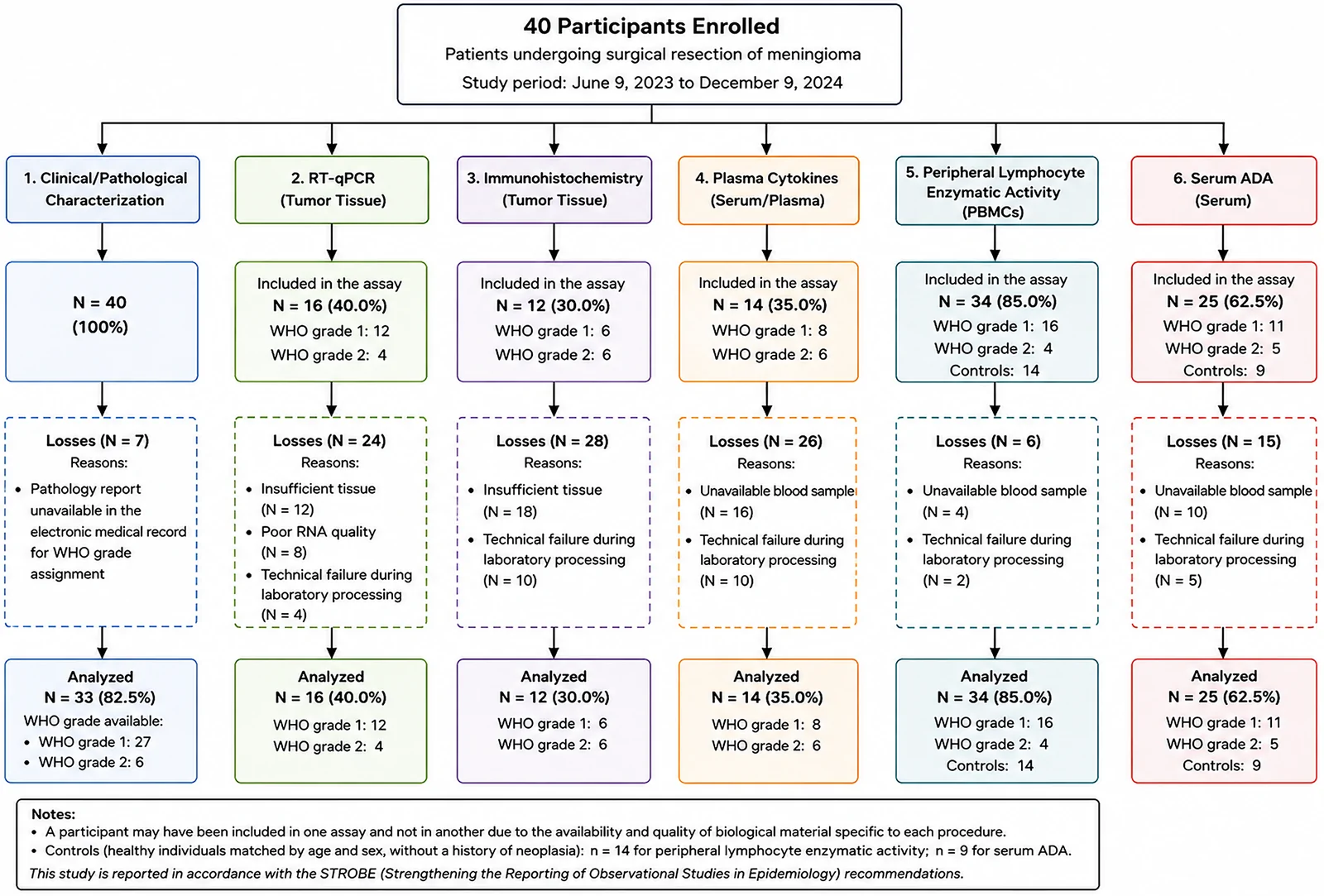
Participant flow and assay-specific sample distribution. Flow diagram showing the 40 enrolled participants, WHO grade availability, assay-specific subsets, reasons for missing samples, and control groups included in the systemic analyses

This cross-sectional, quantitative, unpaired comparative study included patients undergoing surgical resection of meningiomas at Western Regional Hospital (HRO) and Unimed Hospital, Chapecó, Santa Catarina, Brazil, between June 9, 2023 and December 9, 2024. A total of 40 participants with a surgical diagnosis of meningioma were enrolled. WHO grade was available for 33 cases, including 27 WHO grade 1 and 6 WHO grade 2 meningiomas; 7 cases could not be assigned a WHO grade because the pathology report could not be accessed in the electronic medical record. Because the availability and quality of biological material differed across experimental procedures, assay-specific sample sizes varied. RT-qPCR analyses included 12 WHO grade 1 and 4 WHO grade 2, immunohistochemistry included 6 WHO Grade 1 and 6 WHO grade 2, plasma cytokine analyses included 8 WHO grade 1 and 6 WHO grade 2, peripheral lymphocyte enzymatic assays included 16 WHO grade 1, 4 WHO grade 2 and 14 control, and serum ADA analyses included 11 WHO grade 1, 5 WHO grade 2 and 9 control. Reasons for exclusion from each assay included: insufficient tissue, poor RNA quality, unavailable blood sample, and technical failure during laboratory processing. The overlap among assay-specific subsets and the reasons for missing samples are summarized in Fig. 1. Participants were selected by convenience sampling. The control group consisted of 14 individuals without a history of neoplasia, matched to the patient group according to healthy individuals, matched for age and sex. The study is reported in accordance with the STROBE recommendations for observational studies.

### Inclusion and exclusion criteria

Included were individuals diagnosed with meningioma, of both sexes, over 18 years of age, who underwent surgical procedures for tumor removal and were being followed up at referral hospitals. Excluded were patients under 18 years of age, diagnosed with another type of CNS neoplasm, or who underwent other treatment modalities other than surgery. For the control group, healthy individuals over 18 years of age, of both sexes, were included, and those with a history of neoplasia in any site were excluded.

### Collection and processing of biological material

Approximately 20 mL of venous blood were collected and distributed in tubes with sodium citrate, EDTA, and clot activator. The neoplastic tissue sample was collected by the neurosurgeon during resection. The blood was processed to obtain serum and lymphocytes and stored at -80 °C. The tumor tissue was preserved in formalin (for histopathological analysis) and in RNAlater^®^ (for molecular analyses) and frozen at -80 °C.

### Plasma inflammatory profile (flow cytometry)

Plasma concentrations of interferon-gamma (IFN-γ), tumor necrosis factor alpha (TNF-α), interleukin-10 (IL-10), interleukin-6 (IL-6), interleukin-4 (IL-4), and interleukin-2 (IL-2) were determined by flow cytometry using the multiplex Cytometric Bead Array (CBA) Human Th1/Th2 Cytokine Kit II assay (BD Biosciences™). Data acquisition was performed on a BD Accuri™ C6 Plus flow cytometer, and concentrations (pg/mL) were calculated using BD CBA Analysis software (BD Biosciences™, San Diego, CA, EUA – Catalog: 551809).

### Analysis of CD73 and PD-L1 protein expression by immunohistochemistry

The protein expression of cluster of differentiation 73 (CD73) and programmed death-ligand 1 (PD-L1) was evaluated in paraffin-embedded tumor samples. Immunohistochemistry was performed using a commercial Kit (Invitrogen^®^) following fabricant instructions. Deparaffinization and rehydration was performed with xylol, alcohol: 99.8%, 95%, 70%, 30% and distilled water, respectively (5 min each). Then, antigen retrieval was performed with a Tris (10 mM) EDTA (1 mM) solution (pH 9.0) in a water bath at 100 °C for 10 min. Then, the slide tissue was naturally cold. Endogenous peroxidase was blocked using a peroxidase suppressor solution. The slides were incubated overnight with primary antibody anti-CD73 (Abcam ab133582, 1:2000) or anti-PD-L1 antibody (Abcam ab205921, 1:400), respectively, followed by a 30-min incubation with an HRP-conjugated goat anti-rabbit secondary antibody (Invitrogen^®^ 1:2000). Staining was developed using DAB (Dako K3467), and nuclei were counterstained with hematoxylin. Slides were dehydrated, cleared, mounted, and imaged using a Nikon Eclipse H550L microscope with NIS-Elements software. The percentage of areas immunostained for CD73, and PD-L1 was quantified using QuPath software. Negative control was performed without exposure to the tissue from the primary antibody. Briefly, digital images (40x magnification) were opened in QuPath, and immunostaining intensity was evaluated by quantifying the DAB signal profile with a minimum threshold of 0.100. The total percentage of the immunostained area was then determined by analyzing five representative fields from five independent images.

### Gene expression analysis by RT-qPCR

Total RNA was extracted from tumor tissue (~ 50 mg) using TRIzol Reagent, followed by chloroform phase separation, isopropanol precipitation, and washing with 75% ethanol, with the pellet resuspended in RNase-free water. Samples were then treated with DNase I (Ambion/Thermo Fisher kit) to remove contaminating genomic DNA, using 1 µL of DNase I (2 U) per 50 µL reaction containing up to 10 µg of RNA, incubated at 37 °C for 30 min and inactivated with 25 mM EDTA at 65 °C for 10 min. cDNA was synthesized with the High-Capacity cDNA Reverse Transcription Kit (Applied Biosystems/Thermo Fisher), using up to 2 µg of total RNA in a 20 µL reaction containing 10X RT Buffer, 25X dNTP Mix, 10X RT Random Primers, and MultiScribe™ Reverse Transcriptase, following the manufacturer’s recommended cycling conditions. Real-time PCR reactions were performed with PowerUp™ SYBR™ Green Master Mix (Applied Biosystems/Thermo Fisher), in a final reaction volume containing 5 µL of 2X master mix, 0.5 µL of each primer (forward and reverse, final concentration 0.5 µM each), and 20 ng of cDNA template, brought up with nuclease-free water. Amplification was carried out using a two-step standard cycling protocol, consisting of UDG activation at 50 °C for 2 min and polymerase activation (Dual-Lock™) at 95 °C for 2 min, followed by 40 cycles of denaturation at 95 °C for 15 s and combined annealing/extension at 60 °C for 1 min. ACTB was chosen as the reference gene was based on a prior study that assessed the stability of 13 candidate reference genes using geNorm and NormFinder, in which ACTB ranked among the most stable (DOI: https://doi.org/10.1007/s12035-017-0800-3). Quantitative PCR was performed using PowerUp™ SYBR™ Green Master Mix (Applied Biosystems/Thermo Fisher Scientific), with the following primer pairs: ENTPD1/CD39 (forward GCCCTGGTCTTCAGTGTTATTAG; reverse CTGGCATAACCTACCTACTCTTTC), NT5E/CD73 (forward GCCTGGGAGCTTACGATTTTG; reverse TAGTGCCTGGTACTGGTCG), CD274/PD-L1 (forward TGCCGACTACAAGCGAATTACTG; reverse CTGCTTGTTCCAGATGACTTCGG), IL1B/IL-1β (forward GGAGAATGACCTGAGCACCT; reverse GGAGGTGGAGAGCTTTCAGT), ACTB (forward TCCCTGGAGAAGAGCTACG; reverse GTAGTTTCGTGGATGCCACA), and GAPDH (forward CTCCTCACAGTTGCCATGTA; reverse GTTGACACAGGGTACTTTATTG).

Relative gene expression was calculated using the 2^−ΔΔCt method. Relative gene expression was calculated using the 2^−ΔΔCt method. One WHO grade 1 tumor sample was designated as the calibrator and assigned a relative expression value of 1. Target-gene expression was normalized using ACTB as reference gene, and relative fold changes were calculated in relation to the calibrator sample.

### Determination of CD39, CD73, and ADA enzyme activities

The activity of CD39 and CD73 enzymes in lymphocytes was determined according to the protocol of Leal et al. [14]. Lymphocyte isolation was performed using peripheral blood collected in EDTA-containing tubes. Initially, samples were centrifuged at 3,500 revolution per minute (rpm) for 10 min; the plasma was separated for subsequent analysis, and the buffy coat was collected. The buffy coat was diluted with an equal volume of saline solution and homogenized. Next, the cell suspension was carefully layered onto Ficoll-Hypaque at a 1:2 ratio and centrifuged at 1,800 rpm for 30 min to separate the mononuclear cells. The mononuclear cell layer was collected and washed with 10 mL of saline solution, followed by centrifugation at 1,800 rpm for 5 min. After discarding the supernatant, the cell pellet was resuspended in 5 mL of saline solution and centrifuged again at 1,800 rpm for 5 min. When erythrocyte contamination was observed, erythrocyte lysis was performed using a hemolytic buffer based on EDTA and ammonium chloride, followed by centrifugation at 1,000 rpm for 10 min. Subsequently, the cells were washed again with saline solution and centrifuged at 1,800 rpm for 5 min.

CD39 (NTPDase1) ectonucleotidase activity was determined using ATP and ADP as substrates. For each substrate, 20 µL of the cell suspension was added in duplicate to 160 µL of the incubation medium, consisting of 1.2 mol/L NaCl, 600 mM glucose, 50 mM KCl, 500 mM Tris-HCl (pH 8.0), 5 Mm CaCl₂, and distilled water. Subsequently, 20 µL of ATP or ADP was added to separate plates. The samples were incubated at 37°C under agitation for 70 minutes. After incubation, the reaction was stopped by adding 150 µL of 15% trichloroacetic acid (TCA). A 30 µL aliquot of the reaction mixture was transferred to a new plate and combined with malachite green reagent, prepared using malachite green, ammonium molybdate, and polyvinyl alcohol. After 10 minutes for color development, absorbance was measured at 630 nm. The amount of inorganic phosphate released via ATP or ADP hydrolysis was estimated using a phosphate standard curve with concentrations of 5, 10, 20, 30, and 40 nmol. Ecto-5’-nucleotidase/CD73 activity was quantified using the same procedure, changing only the substrate. Enzymatic activity was calculated based on the mean inorganic phosphate released in duplicate samples during the incubation period, adjusted by a correction factor derived from the sample volume and protein concentration, and expressed as nmol Pi/min/mg protein.

Adenosine deaminase (ADA) activity was determined in serum samples using the method of Giusti [15]. To obtain the serum, the blood collected in a tube containing a clot activator was subjected to centrifugation at 3,500 rpm for 15 min. The assay is based on the quantification of ammonia released during the deamination of adenosine to inosine, a reaction catalyzed by ADA. For the analysis, 30 µL of serum was incubated with a 21 mM adenosine solution (prepared in 50 mM phosphate buffer, pH 6.5) at 37 °C for 60 min. Following incubation, phenol/sodium nitroprusside and alkaline hypochlorite solutions were added to form an indophenol complex; the intensity of the resulting color is proportional to the amount of ammonia produced during the enzymatic reaction. The samples were then incubated again at 37 °C for 30 min with agitation. Absorbance was measured spectrophotometrically at 620 nm, and a blank was prepared by substituting the sample with ultrapure water. ADA activity was calculated based on the difference between the sample absorbance and the sample blank absorbance, using a calibration factor derived from an ammonium sulfate standard curve, and was expressed in U/L.

### Statistical analyses

Data normality was tested using the Shapiro-Wilk test. Non-parametric results were expressed as median and interquartile range. Outliers were identified by the Grubbs test. For cytokines, RT-qPCR and immunohistochemistry analysis the Mann-Whitney test was used. CD39, CD73 and ADA activity in lymphocytes of patients with meningiomas were analyzed by Kruskal-Walli’s test. Regarding enzymatic activity, multiple comparisons were performed to compare all groups, including control and meningiomas grade 1 and grade 2. To control the false discovery rate (FDR) arising from multiple comparisons, *p*-values were adjusted using the Benjamini-Hochberg procedure. Differences were considered statistically significant when *p* ≤ 0.05. To investigate the possible correlation among purinergic signaling alterations CD73 with other molecular inflammatory and immunosuppressive endpoints e.g. PD-L1 and IL-1β a Spearman correlation was performed. The analyses were performed using GraphPad Prism 9.0 software.

## Results

During the data collection period, 40 patients diagnosed with meningioma underwent surgical resection. Of these, 6 patients were lost to follow-up due to histopathological results and were not included in the marker analyses, except for the analysis of the mean age of the total group (*n* = 40). The mean age of the 40 patients was 52.9 years (± 14.7). There was a predominance of females (85%; *n* = 34), with a ratio of 5.7:1 women for every man. Regarding the WHO 2021 classification, 67.5% (*n* = 27) were classified as WHO grade 1, 15% (*n* = 6) as WHO grade 2, and 17.5% (*n* = 7) did not have a grade reported. The age group most affected in WHO grade 1 meningiomas was between 51 and 64 years (39.2%), while in WHO grade 2, the age group above 65 years predominated (66.6%). A higher incidence of WHO grade 2 tumors was observed in patients over 65 years of age (*p* = 0.0180). Systemic arterial hypertension was the most prevalent comorbidity, affecting 42.8% of patients with WHO grade 1 and 66.6% of WHO grade 2. Regarding neurological symptoms, 27.5% of patients (*n* = 11) presented with seizures in the preoperative period. Persistent headache was the reason for diagnostic investigation in 75% of cases, and 80% of patients did not present with focal neurological deficits at diagnosis. The preoperative Karnofsky Performance Scale score was 80–100 in 77.5% of patients. The predominant tumor location was the frontal convexity (65%). Among patients with available information on extent of resection, complete resection was documented in 35/40 (87,5%) patients. The denominator excludes one patient for whom resection status was unavailable (Table 1).

**Table 1:** Sample characterization

**Sex**		**History of Persistent Headache**	
Female	34 (85%)	Yes	30 (75%)
Male	6 (15%)	No	10 (25%)
**Age**		**Presence of Seizures**	
21–30 years	5 (12,5%)	No	29 (72.5%)
31–40 years	1 (2,5%)	Yes	11 (27.5%)
41–50 years	12 (30%)	**Use of Anticonvulsant Medication**	
51–60 years	11 (27.5%)		
61–70 years	5 (12,5%)	No	29 (72.5%)
> 71 years	6 (15%)	Yes	11 (27.5%)
**WHO Classification (2021)**		KPS Scale Score **	
WHO Grade 1	27 (67.5%)	80–100	31 (77.5%)
WHO Grade 2	6 (15%)	50–70	8 (20%)
Not reported	7 (17.5%)	0–40	1 (2.5%)
**Predominant Location**		**Laterality**	
Frontal convexity	26 (65%)	Left	17 (42.5%)
Parietal convexity	8 (20%)	Right	14 (35%)
Posterior fossa	5 (12.5%)	Anterior fossa	7 (17.5%)
Temporal convexity	1 (2.5%)	Posterior fossa	2 (5%)
Tumor Volume*		**Neurological Deficit at Diagnosis**	
1–20	22 (55%)		
21–40	8 (20%)	No deficit	32 (80%)
41–60	5 (12.5%)	Motor	6 (15%)
61–80	3 (7,5%)	Language	1 (2.5%)
> 100	2 (5%)	Mixed	1 (2.5%)
**Extent of Surgical Resection**		Preoperative Corticosteroid Use***	
Total	35 (87.5%)	No	28 (70%)
Subtotal	3 (7.5%)	Yes (8 mg/day)	9 (22.5%)
Supratotal	1 (2.5%)	Yes(12 mg/day)	2 (5%)

* Preoperative magnetic resonance imaging estimate (cm³ = mL)

** Karnofsky Performance Status (KPS). *** Dexamethasone - total daily dose

### Systemic inflammatory profile (plasma)

**Fig. 2:**
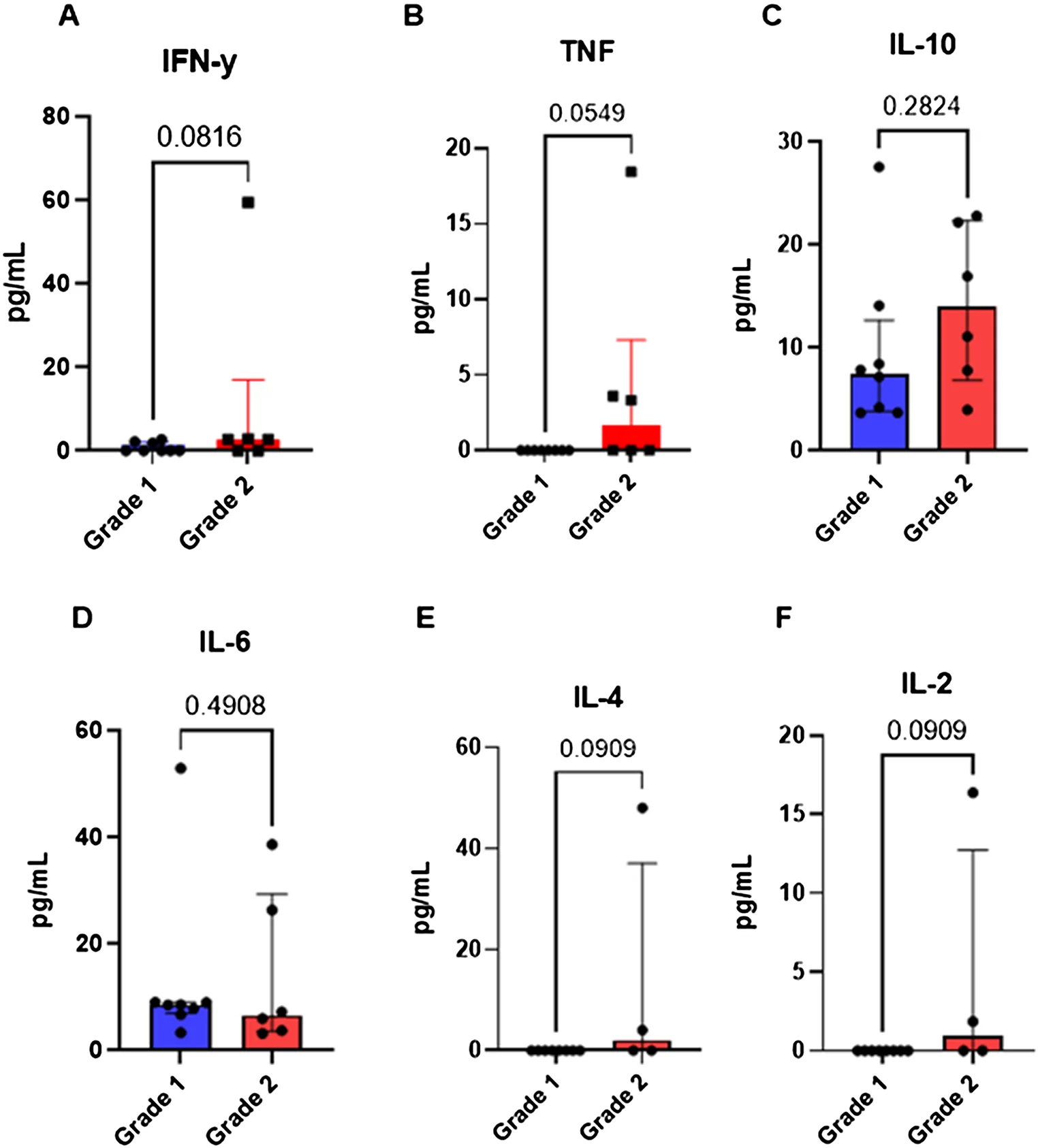
Plasma inflammatory profile in WHO grade 1 (*n* = 8) and WHO grade 2 (*n* = 6) meningiomas. (**A**) IFN-γ, (**B**) TNF. (**C**) IL-10. (**D**) IL-6. (**E**) IL-4. (**F**) IL-2. Data are expressed as median and interquartile range. Results were analyzed by Mann-Whitney test. Differences were considered statistically significant when *p* ≤ 0.05

Analysis of the inflammatory profile in plasma of patients with meningiomas showed a tendency towards an increase in the average concentration of all six cytokines analyzed IFN-γ (*U* = 11; *p* = 0.08; Fig. 2A); TNF (*U* = 12; *p* = 0.054; Fig. 2B); IL-10 (U = *p* = 0.28; Fig. 2C); IL-6 (*U* = 15; *p* = 0.49; Fig. 2D); IL-4 (*U* = 8; *p* = 0.09; Fig. 2E); IL-2 (*U* = 8; *p* = 0.09; Fig. 2F) in the group of patients with WHO grade 2 meningiomas compared to WHO grade 1, although without statistical significance.

### Genic expression in the tumor microenvironment (RT-qPCR)

#### Analysis of CD39, CD73, PD-L1, and IL-1β

**Fig. 3:**
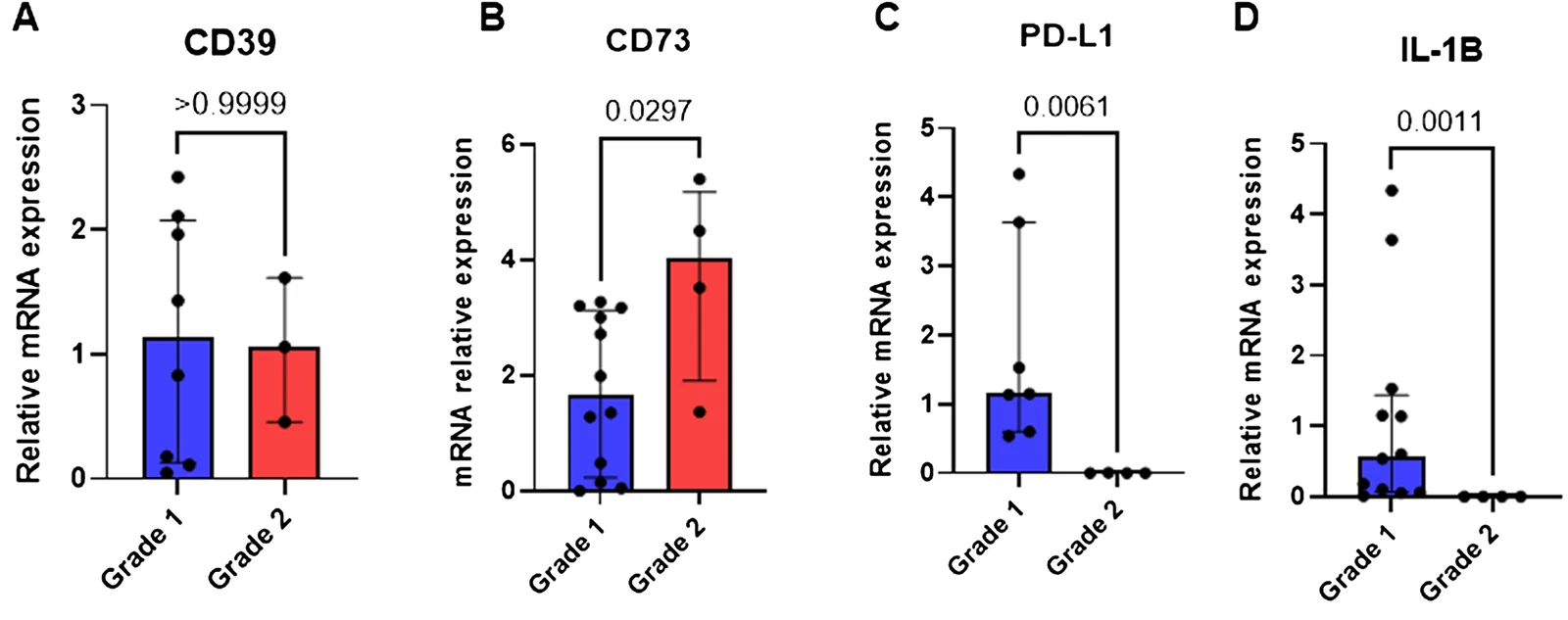
Gene expression profile in WHO grade 1 (*n* = 8–12) and WHO grade 2 (*n* = 3–4) meningiomas. (**A**) CD39 mRNA expression. (**B**) CD73 mRNA expression. (**C**) PD-L1. (**D**) IL-1β mRNA expression. Data are expressed as median and interquartile range. Results were analyzed by Mann-Whitney test. Differences were considered statistically significant when *p* ≤ 0.05

RT-qPCR analyses were performed in assay-specific subsets according to the availability of technically valid measurements. For CD73, significantly higher expression was observed in WHO grade 2 meningiomas compared to WHO grade 1, with an increase of approximately 2.15 times (Fold Change) (*U* = 6; *p* = 0.0297; CI = 95.82%: 0.08 to 4.11) (Fig. 3B). Conversely, the tumor microenvironment of patients with WHO grade II exhibited reduced PD-L1 expression (*U* = 0; *p* = 0.006; CI = 95.76%: 4.334 to -0,5396) (Fig. 3C); and reduced IL-1β relative mRNA expression when compared to WHO grade 1 meningiomas (*U* = 0; *p* = 0.0011; CI = 95.82%: 0,348 to -0,003) (Fig. 3D). CD39 mRNA did not change among meningioma grade 1 and 2 groups (*U* = 9; *p* > 0.999) (Fig. 3A). To investigate if changes in purinergic signalling were positive or negative correlated with other molecular alterations a Spearman correlation was performed. However, no differences were found in Spearman’s correlation (Table 2).

### Protein expression in the tumor microenvironment (immunohistochemistry)

**Fig. 4:**
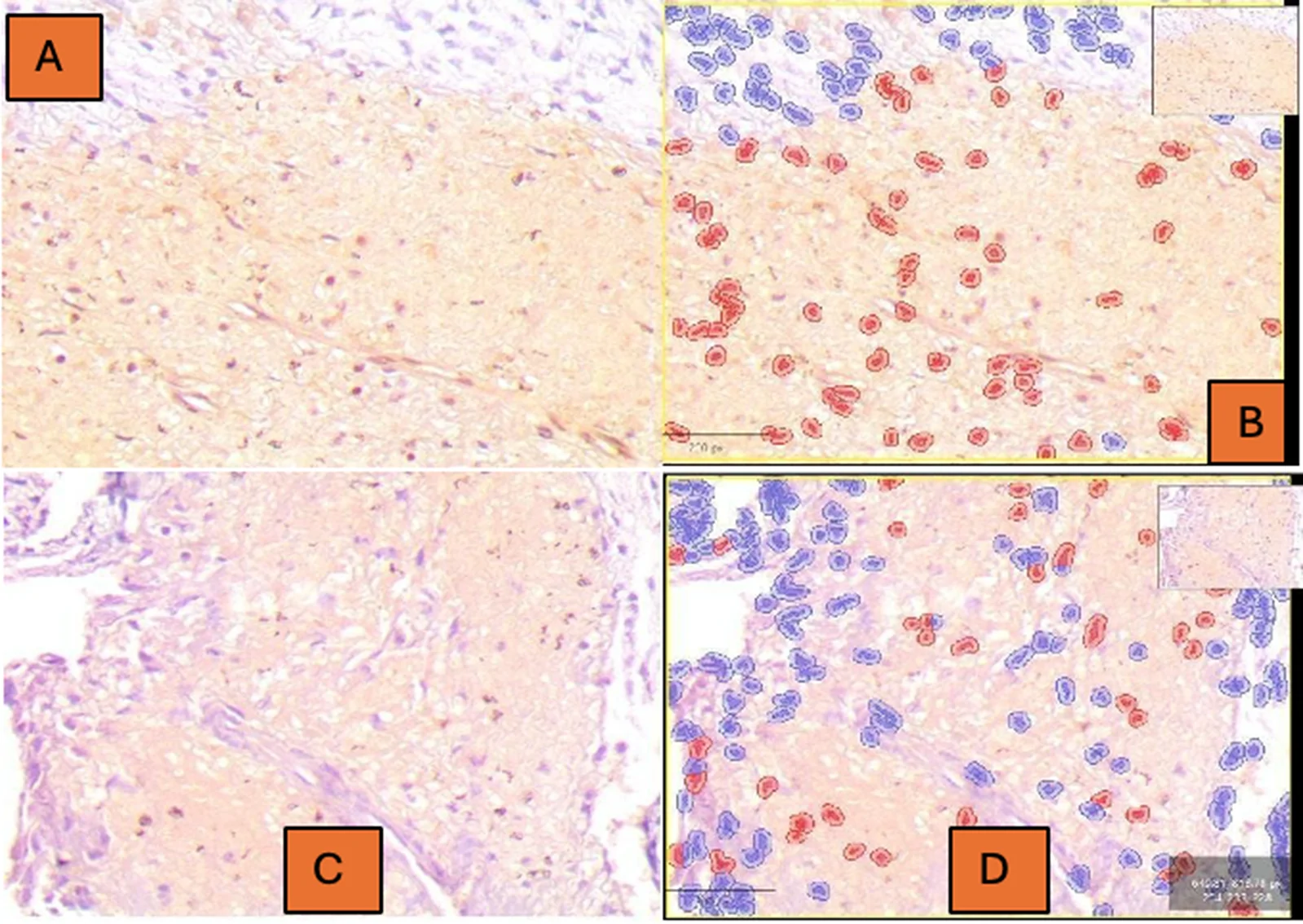
CD73 - In **A**: Digitized slide. **B**: Graphical analysis of the slide using QuPath software. PDL-1 - In **C**: Digitized slide. **D**: Graphical analysis of the slide using QuPath software

**Fig. 5:**
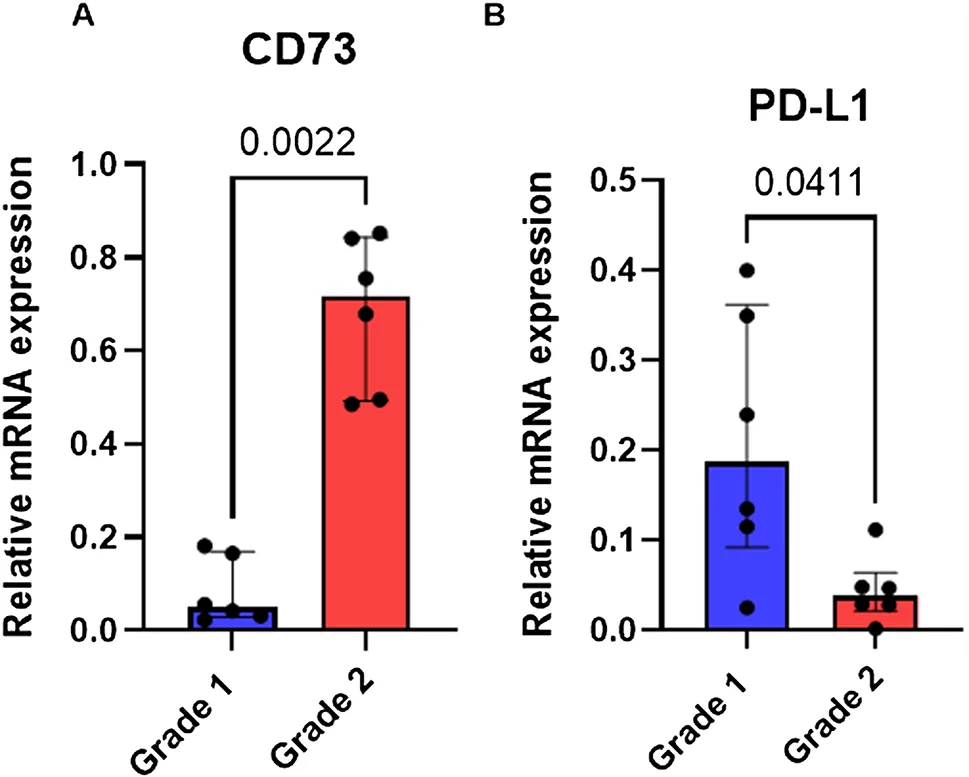
Immunohistochemistry analysis of CD73 and PD-L1 protein expression in WHO grade 1 and WHO grade 2 meningiomas. WHO grade 2 tumors (*n* = 6), WHO grade 1 tumors (*n* = 6). Data are expressed as median and interquartile range. Results were analyzed by Mann-Whitney test. Differences were considered statistically significant when *p* ≤ 0.05

Immunohistochemistry analysis revealed that CD73 and PD-L1 are different expressed in meningioma tumor tissues WHO grade 1 and WHO grade 2 (Fig. 4). Protein analysis yielded significantly higher CD73 expression in WHO grade 2 meningiomas compared to WHO grade 1 (*U* = 0; *p* = 0.0022; CI 95.89%: 0.44 to 0.7) (Fig. 5A). In addition, higher PD-L1 expression in WHO grade 1 meningiomas compared to WHO grade 2 (*U* = 5; *p* = 0.0411; CI 95.89%:-0,34 to -0.003) (Fig. 5B).

**Table 2:** Correlation between CD73 and PD-L1, and IL-1β expression

Group	Genic/protein expression parameters			
**Meningiomas WHO Grade 1**	**PD-L1**		**IL-β**	
	*r*	*p*	*r*	*p*
CD73 mRNA expression	0.1429	0.3760	-0.3427	0.1381
CD39 mRNA expression	0.0476	0.9349	0,6429	0.0962
CD73 protein expression (IHQ)	-0.3000	0.3417	-	-
**Meningiomas WHO Grade 2**	**PD-L1**		**IL-β**	
	*r*	*p*	*r*	*p*
CD73 mRNA expression	-0.8000	0.3333	0.2000	0.2000
CD39 mRNA expression	0.5000	> 0.999	-0.5000	> 0.999
CD73 protein expression (IHQ)	0.5429	0.1486	-	-

Data are analyzed by Spearman correlation test. Spearman’s correlation coefficient (*r*) and p values are shown. (*n* = 8–12, WHO grade 1; *n* = 3–4, WHO grade 2)

### Enzymatic activity in lymphocytes

#### Lymphocytes

**Fig. 6:**
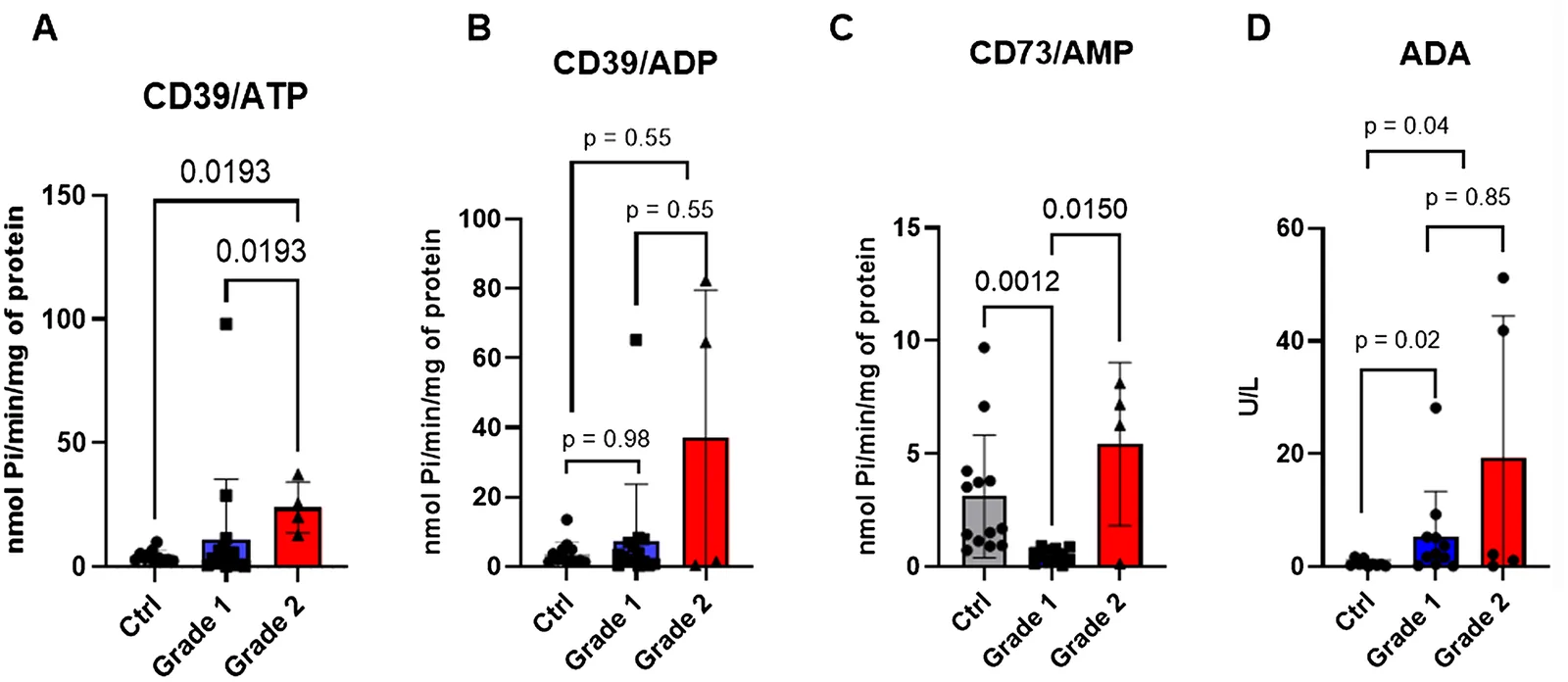
Enzymatic activity of CD39, CD73 and adenosine deaminase in lymphocytes from WHO grade 1 and WHO grade 2 meningiomas and in controls. (**A**) CD39 that hydrolyzes ATP. (**B**) CD39 that hydrolyzes ADP. (**C**) CD73 that hydrolyzes AMP. (**D**) ADA that hydrolyzes adenosine in inosine. Data are expressed as median and interquartile range. Results were analyzed by Kruskal-Wallis test. The FDR method of multiple comparisons was applied when appropriated. Differences were considered statistically significant when *p* ≤ 0.05. (*n* = 11; WHO grade 1; *n* = 4–5 WHO grade 2; *n* = 9–13 control)

The Fig. 5 shows enzymatic activity of CD39, CD73 and ADA in lymphocytes of patients diagnosed with meningiomas WHO grade 1 and WHO grade 2 as well as control group. Kruskal-Wallis, revealed that CD39 activity in ATP hydrolysis was significantly higher in WHO grade 2 compared to the control (*H* = 6.52; *p* = 0.01; Fig. 6A) and between grades (WHO grade 1 vs. WHO grade 2; *p* = 0.01). There was no significant difference in ADP hydrolysis comparing control and meningiomas (Fig. 6B). CD73 activity in AMP hydrolysis was increased in WHO grade 2 compared to WHO grade 1 (*H* = 14.5; *p* = 0.01) and between control and WHO grade 1 (*p* = 0.0012) (Fig. 6C). Importantly, ADA activity was increased in patients WHO grade 1 (*H* = 6.52; *p* = 0.02) and WHO grade 2 compared with control (*H* = 6.52; *p* = 0.04; Fig. 6D). However, ADA activity did not differ between meningiomas grades.

## Discussion

The sample characterization showed a predominance of females (85%), with a ratio of 12.3 women for every man, higher than that described in the literature, which indicates a ratio of 2:1. This discrepancy may reflect specific characteristics of the studied population, regional prevalence variables, the influence of hormonal factors, or differences in cases referred to the service. In WHO grade 2 meningiomas, approximately 50% of the sample was composed of men, diverging from previous studies. This difference may occur due to variations among the sample populations regarding geographic location, modifiable health factors, and genetics. The average age was 52.9 years, with a standard deviation of 14.7 years. According to Szulzewsky et al. [2], meningiomas are significantly more frequent in individuals over 65 years of age. In this study, it was observed that 66.6% of WHO grade 2 meningiomas occurred in individuals aged 65 years or older (*p* = 0.0180). Systemic arterial hypertension was the most frequent comorbidity, affecting 42.8% of WHO grade 1 cases and 66.6% of WHO grade 2 cases.

Regarding occupation, 13.3% of the sample consisted of patients whose work activity was agriculture, which may be related to tumor development, since large-scale agriculture, livestock farming, and documented exposure to pesticides are associated with an increased risk of CNS tumors. In the study by Petit et al. [16], an increased risk ratio of 1.71 for meningioma development among farmers engaged in mixed beef and dairy cattle farming. Therefore, further research would be valuable to determine whether a causal relationship exists by evaluating the amount of exposure, duration, effects, and direct comparisons with other occupational activities.

Plasma cytokine analysis showed a non-significant trend toward higher concentrations of IFN-γ, TNF, IL-10, IL-6, IL-4, and IL-2 in patients with WHO grade 2 meningiomas compared with WHO grade 1 tumors. TNF showed the closest approach to the predefined significance threshold (*p* = 0.0549), followed by IFN-γ (*p* = 0.0816). These findings should be interpreted cautiously because of the small sample size (WHO grade 1, *n* = 8; WHO grade 2, *n* = 6), substantial interindividual variability, and limited statistical power. Although IL-4 and IL-10 are biologically implicated in pathways related to macrophage polarization and immune regulation, the present study did not assess intratumoral macrophage phenotypes and therefore cannot establish an association with M2 polarization. Similarly, no individual-level analysis was performed between cytokine concentrations and seizure status; consequently, the cytokine findings should not be interpreted as clinically associated with seizures. These exploratory trends warrant independent validation in larger cohorts incorporating paired clinical and tumor immune-phenotyping data [17–22]. 

Regarding NT5E/CD73 expression in tumor tissue, a statistically significant increase was observed in WHO grade 2 meningiomas compared with WHO grade 1 tumor (*p* = 0.0297), corresponding to an approximately 2.15-fold difference. This finding identifies CD73 as a candidate biomarker associated with WHO grade in the present cohort. CD73 is the ecto-5′-nucleotidase responsible for converting extracellular AMP into adenosine, a metabolite with well-established immunoregulatory properties in several tumor contexts [8, 23–25]. Accordingly, increased CD73 expression in WHO grade 2 tumors provides biological rationale for investigating whether adenosinergic signaling differs between meningioma grades. However, extracellular adenosine concentrations, adenosine receptor expression, and downstream signaling were not directly measured in this study. Therefore, the present findings do not demonstrate CD73-driven immunosuppression or immune escape and should instead be regarded as evidence supporting a testable mechanistic hypothesis for future investigation.

In contrast to CD73, PD-L1 gene expression was drastically reduced in WHO grade 2 meningiomas compared to WHO grade 1 (*p* = 0.0134). IL-1β gene expression was significantly higher in WHO grade I compared to WHO grade 2 (*p* = 0.0011). Protein analysis by immunohistochemistry validated the transcriptional findings, revealing a statistically significant dichotomy: CD73 expression was markedly higher in WHO grade 2 meningiomas compared to WHO grade 1 (*p* = 0.0022), with averages of 0.684 (± 0.163) and 0.082 (± 0.071), respectively. Conversely, PD-L1 immunostaining was higher in WHO grade 1 tumors compared to WHO grade 2 tumors (*p* = 0.0411), with averages of 0.211 (± 0.145) and 0.044 (± 0.037).

This data suggests the existence of a post-transcriptional regulatory mechanism acting on the progression of meningiomas. The high presence of PD-L1 mRNA in WHO grade 2 tumors indicates that the gene is being actively transcribed, but the absence of the corresponding protein suggests that its translation is being blocked or that the protein is being degraded. This phenomenon, known in the oncology literature as mRNA-protein discordance, points to the existence of complex post-transcriptional regulatory mechanisms acting on the progression of meningiomas. The low expression of PD-L1 in higher-grade meningiomas questions the effectiveness of monotherapy with checkpoint inhibitors for high-grade disease [26, 27].

The lower PD-L1 expression observed in WHO grade 2 meningiomas in the present cohort differs from the findings reported by Du et al. [26], who described increased PD-L1 expression in anaplastic meningiomas, currently classified as WHO grade 3 tumors. Several factors may account for this apparent discrepancy. First, WHO grade 2 and WHO grade 3 meningiomas represent biologically distinct disease categories, and findings from grade 3 tumors cannot be directly extrapolated to grade 2 diseases. Second, differences in cohort composition, tissue processing, antibody clones, staining protocols, positivity thresholds, and scoring strategies may substantially influence PD-L1 immunohistochemical measurements. In addition, PD-L1 expression by neoplastic cells is biologically distinct from PD-L1 expression by infiltrating immune or stromal cells, and differences in the relative abundance of these compartments may affect both bulk transcript measurements and tissue-level immunohistochemical estimates. Thus, rather than establishing a grade-dependent switch in immune-evasion mechanisms, the divergent findings highlight the need for standardized, compartment-resolved analyses of PD-L1 across WHO grades in larger meningioma cohorts [28].

Although CD73 is under investigation as a therapeutic target in several malignancies, the present study did not evaluate treatment response, recurrence, progression-free survival, overall survival, or the functional consequences of CD73 inhibition. Therefore, our findings do not establish CD73 as a therapeutic determinant in meningioma. Rather, the grade-associated increase in CD73 expression provides a rationale for future studies evaluating its prognostic and therapeutic relevance.

In the systemic analysis of lymphocytes, CD39 activity regarding in ATP hydrolysis was significantly higher in WHO grade 2 cases compared to the control (*p* = 0.0386) and across WHO grades (*p* = 0.0212), this differs from in situ findings due to the differences in methodological approaches and the biological compartments analyzed. This increased activity may grant WHO grade 2 tumors a greater capacity to hydrolyze extracellular ATP into AMP. Adenosine can exert immunomodulatory effects and contribute to mechanisms associated with tumor progression in various neoplasms with systemic effects. Regarding the hydrolysis of ADP into AMP, no statistically significant difference was observed, which may indicate distinct regulation of this product by CD39. This increased activity may give WHO grade 2 a greater capacity for hydrolysis extracellular ATP into AMP. Adenosine can exert immunomodulatory effects and contribute to mechanisms associated with tumor progression in various neoplasms with systemic effects. Regarding the hydrolysis of ADP into AMP, no statistically significant difference was observed, which may indicate distinct regulation of this product by CD39. Furthermore, an increase in CD73 activity was observed in lymphocytes from patients with WHO Grade 2 meningiomas compared to those with WHO Grade 1 (*p* = 0.0299), possibly demonstrating the effect of the purinergic axis. CD73 and its product, adenosine, influence the generation of various tumors through mechanisms involving cell proliferation, and therapeutic resistance [8, 23, 24].

An important point to highlight is the increased CD73 expression rates in the control group when compared to WHO grade 1, suggesting that purinergic activity may be elevated in basal or non-neoplastic conditions [29]. According to Allard et al. [7], physiologically, CD73 and CD39 act as functional regulators of anti-inflammatory and immunosuppressive action during inflammation. These processes occur even without neoplastic involvement potentially associated with chronic conditions such as diabetes, hypertension, and obesity, which are common in the general population and may influence the findings observed across tumor grades, particularly given the small sample size.

In the serum ADA analysis, there was no statistically significant difference between the groups; however, a 76.8% increase was observed in WHO grade 2 compared to the control and a 62.5% increase in WHO grade 1 compared to the control. This percentage increase suggests moderate activation of the ADO degradation pathway. The literature demonstrates that ADA expression is highly dependent on tumor type and the biological pattern of the lesion, which may explain the lack of statistical significance associated with sample variability [23, 24, 30, 31].

When considered together, the tissue and systemic findings suggest grade-associated differences across distinct biological compartments, but they should not be integrated as evidence of a single pathophysiological mechanism. The increased NT5E/CD73 expression observed in WHO grade 2 tumor tissue and the differences in peripheral ectonucleotidase activity constitute convergent observations that support further investigation of purinergic signaling in meningioma biology. Nevertheless, peripheral lymphocyte enzyme activity and circulating cytokine concentrations do not necessarily reflect purinergic activity or immune-cell behavior within the tumor microenvironment. Moreover, extracellular adenosine concentrations, adenosine receptor signaling, intratumoral immune-cell phenotypes, and functional consequences of CD73 activity were not directly assessed. Interpretation is further limited by the cross-sectional design, small and assay-specific sample sizes, tumor heterogeneity, and potential systemic confounders such as age, comorbidities, corticosteroid exposure, and other medications. Accordingly, adenosine-mediated immunosuppression and changes in immune-cell composition should be considered biologically plausible hypotheses generated by the present findings rather than mechanisms demonstrated by this study. Future longitudinal studies combining compartment-resolved tissue analysis, direct measurement of adenosine signaling, immune phenotyping, and clinically relevant outcomes will be required to determine the biological and clinical significance of these observations.

## Limitations

This study presents certain limitations that should be considered when interpreting the findings. First, the cross-sectional design and small sample size limit the ability to establish causality between the observed alterations and the development or progression of meningiomas; furthermore, such a design may exhibit greater instability and be influenced by the participants’ individual characteristics. The inclusion of only four individuals with WHO grade II tumors who underwent resection during the study period also reduced the group’s statistical representativeness, meaning potential grade-related differences may have gone undetected. Additionally, there was a loss to follow-up regarding six histopathological results in the health units’ electronic medical records, preventing their inclusion in the analyses. In the systemic analyses, confounding factors—such as arterial hypertension and medication use—may have influenced inflammatory markers and purinergic system components. Similarly, the use of corticosteroids to manage tumor-associated symptoms can interfere with immune responses and the obtained results. The smaller number of blood samples relative to the total sample size, resulting from logistical constraints during the study’s setup, also limits the comparison of systemic findings. Further studies are needed to better elucidate how purinergic system components function in meningiomas. Additionally, future research would benefit from a larger sample size for systemic analysis, rigorous control of variables such as comorbidities and medication use, and the inclusion of clinical indications beyond just surgical intervention. Exploring purinergic system parameters in meningiomas yields greater insight into the tumor’s biological mechanisms, which is essential for understanding its development and progression. This research is expected to stimulate the development of further studies exploring this pathway, assessing its viability for creating new therapeutic approaches for this neoplasm, which is highly prevalent in the central nervous system [8, 32, 33].

## Conclusion

The clinical-epidemiological characterization of patients with meningiomas undergoing surgical resection in the western region of Santa Catarina revealed a female predominance and a higher frequency of higher-grade tumors among older individuals. Systemic arterial hypertension was the most prevalent comorbidity. The need to consider occupational factors that may increase the risk of developing and progressing these tumors was evident, particularly regarding agricultural activities, necessitating further research. The predominant tumor location was the frontal convexity, and complete resection was achieved in the majority of cases. The preoperative Karnofsky Performance Scale score indicated high functionality in most patients prior to surgery.

In this cross-sectional cohort of surgically treated meningiomas, analyses restricted to WHO grade 1 and WHO grade 2 tumors identified distinct grade-associated molecular patterns. NT5E/CD73 mRNA and CD73 protein expression were higher in WHO grade 2 tumors, whereas CD274/PD-L1 mRNA and PD-L1 protein expression were lower. Differences in peripheral CD39/CD73 enzymatic activity were also observed among the study groups, while circulating cytokines showed non-significant trends and should be considered exploratory.

These findings support an association between CD73- and PD-L1-related profiles and WHO grade but do not establish tumor progression, a transition between immune-evasion mechanisms, or adenosine-mediated immunosuppression. Given the cross-sectional design, small assay-specific sample sizes, absence of WHO grade 3 molecular comparisons, lack of longitudinal clinical outcomes, and absence of direct assessment of adenosine signaling, the results should be considered hypothesis-generating. Nevertheless, the consistent increase in CD73 expression at the mRNA and protein levels in WHO grade 2 tumors identifies CD73 as a candidate grade-associated biomarker deserving further investigation. Validation in larger prospective cohorts, together with cell-type-resolved and functional studies, will be necessary to determine its biological, prognostic, and potential therapeutic relevance in meningiomas.

## Data Availability

No datasets were generated or analysed during the current study.
